# Protein C Inhibitor (PCI) Binds to Phosphatidylserine Exposing Cells with Implications in the Phagocytosis of Apoptotic Cells and Activated Platelets

**DOI:** 10.1371/journal.pone.0101794

**Published:** 2014-07-07

**Authors:** Daniela Rieger, Alice Assinger, Katrin Einfinger, Barbora Sokolikova, Margarethe Geiger

**Affiliations:** 1 Department of Vascular Biology and Thrombosis Research, Center for Physiology and Pharmacology, Medical University of Vienna, Vienna, Austria; 2 Department of Physiology, Center for Phsiology and Pharmacology, Medical University of Vienna, Vienna, Austria; National Institute of Biological Sciences, Beijing, China

## Abstract

Protein C Inhibitor (PCI) is a secreted serine protease inhibitor, belonging to the family of serpins. In addition to activated protein C PCI inactivates several other proteases of the coagulation and fibrinolytic systems, suggesting a regulatory role in hemostasis. Glycosaminoglycans and certain negatively charged phospholipids, like phosphatidylserine, bind to PCI and modulate its activity. Phosphatidylerine (PS) is exposed on the surface of apoptotic cells and known as a phagocytosis marker. We hypothesized that PCI might bind to PS exposed on apoptotic cells and thereby influence their removal by phagocytosis. Using Jurkat T-lymphocytes and U937 myeloid cells, we show here that PCI binds to apoptotic cells to a similar extent at the same sites as Annexin V, but in a different manner as compared to live cells (defined spots on ∼10–30% of cells). PCI dose dependently decreased phagocytosis of apoptotic Jurkat cells by U937 macrophages. Moreover, the phagocytosis of PS exposing, activated platelets by human blood derived monocytes declined in the presence of PCI. In U937 cells the expression of PCI as well as the surface binding of PCI increased with time of phorbol ester treatment/macrophage differentiation. The results of this study suggest a role of PCI not only for the function and/or maturation of macrophages, but also as a negative regulator of apoptotic cell and activated platelets removal.

## Introduction

Protein C Inhibitor (PCI) is a serine protease inhibitor belonging to the class of serpins [1]. Initially PCI has been described as an inhibitor of the vitamin K dependent anticoagulant protease activated protein C (APC) [2]. In the course of further research PCI revealed broad protease reactivity and was acknowledged as inhibitor of different coagulation factors, like thrombin, factor Xa and XIa, but also of fibrinolytic enzymes, uncovering PCI as a balancing factor in hemostasis [3], [4]. Synthesized mainly in the liver, human PCI circulates in plasma at a concentration of about 5µg/ml (approx. 100 nM). Besides that the serpin is synthesized by many cell types and tissues and is present in almost every body fluid. In order to define new markers for myocardial or thrombotic diseases, PCI and the PCI-APC complex have been frequent targets of interest. Indeed elevated blood plasma levels of PCI were detected in male survivors of myocardial infarction [5] and high APC-PCI levels are associated with higher early death rates after aortic surgery [6]. Watanabe et al. [7] found higher APC-PCI complex levels in patients suffering from disseminated intravascular coagulation, thrombotic thrombocytopenia, acute myocardial infarction, pulmonary embolism, and deep vein thrombosis. Decreased PCI levels were found in disseminated intravascular coagulation and thrombotic thrombocytopenia, showing the importance but also the complexity of the APC-PCI balance in coagulation and several thrombotic disorders [7].

Binding to certain glycosaminoglycans, like heparin stimulates the inhibitory activity of PCI for certain target proteases [8], [9]. Heparin binding increases the rate of APC inhibition by PCI about 400 fold [10] and thrombin inhibition 30 fold [11], [12]. PCI also binds oxidized and unoxidized negatively charged phospholipids like phosphatidylethanolamine (PE) and phosphatidylserine (PS), which also modulate its inhibitory activity [13].

These phospholipids are normally present on the inner leaflet of the plasma membrane. PS becomes surface exposed during apoptosis and cell activation. In apoptosis PS serves as an important marker and phagocytosis signal [14], [15]. The phagocyte interacts either directly with PS via a receptor [16], [17], or via a linking molecule, opsonizing PS before the docking of the phagocyte. Milk fat globule –EGF-factor-8 and protein S are well known opsonins, stimulating phagocytosis of apoptotic cells (efferocytosis) acting as a bridge for the exposed PS [18], [19]. Current literature also reveals PS-binding proteins that inhibit phagocytosis of apoptotic cells, like the well known apoptosis marker Annexin V [20], or high mobility group protein-1 [21]. Data obtained in the study by Malleier et al. [13], revealing PS as a binding partner of PCI, were performed in a purified system, not taking into account the influence of plasma membranes. It was therefore our aim to analyse binding of PCI to phospholipds on cell membranes, and to determine whether there is a difference in PCI binding to apoptotic and activated cells, which expose a higher percentage of PCI-binding phospholipids on their surface in comparison to quiescent cells.

## Materials and Methods

### Cell culture and differentiation

Human myeloid U937 cells, human monocytic THP-1 cells (collections of the Department of Vascular Biology and Thrombosis Research, Medical University of Vienna, purchased from ATCC, American Type Culture Collection, Manassas, VA, USA) [22], [23] and human Jurkat cells (a kind gift of Prof. Veronika Sexl, purchased from ATCC, American Type Culture Collection, Manassas, VA, USA) [24], a T-lymphocyte cell line, were cultured in RPMI medium supplemented with 10% FBS (Sigma-Aldrich, St.Luis, MO, USA), 1% Penicillin/Streptomycin/Fungizone, 1% L-glutamine and 2% HEPES (Lonza, Basel, Switzerland), defined as full medium. All cells were maintained in a humidified atmosphere of 5% CO_2_ and a temperature of 37°C. For differentiation U937 cells were seeded at 5×10^5^ cells/ml into 24-well plates (Nunc, Thermo Fisher Scientific, Waltham, MA, USA) and treated for 24 h or 72 h with 20 nM PMA (Sigma-Aldrich, St.Luis, MO, USA) [25], [26]. THP-1 cells were differentiated with 1µM PMA for 24 h at 37°C and 5% CO_2_. Jurkat cells were driven into apoptosis by treatment with 6µM Camptothecin (Sigma-Aldrich, St.Luis, MO, USA) at a density of 1×10^6^ cells/ml in full medium for 24 h (Ct cells). Apoptosis was determined by Annexin V-EGFP or -FITC/propidium iodide staining (Apoptosis kit, Biovision, Milpitas, CA, USA) according to the manufacturer's protocol and analysed by FACS (FACS Calibur, Becton Dickinson, Franklin Lakes, NJ, USA).

Isolation and maintenance of platelets and monocytes from human blood was performed as described by Badrnya et al.[27]. In brief, healthy blood donors gave their informed consent and experiments were approved by the Human Ethics Committee of the Medical University of Vienna, project number EK112/2009. Platelets isolation was performed with citrated blood by two centrifugation steps, 125×g for 20 minutes and 3000×g for 2 minutes on the platelet rich fraction with the addition of prostacyclin (PGI2) (Sigma-Aldrich, St.Luis, MO, USA) at a concentration of 1µM. Monocytes were isolated from blood samples anticoagulated with ethylenediaminetetraacetic acid (EDTA). Peripheral mononuclear blood cells were obtained by density centrifugation with Histopaque 1077 and Histopaque 1119 (Sigma-Aldrich, St.Luis, MO, USA). On the whole, experiments were performed with blood samples, drawn from 15 healthy individuals.

### PCI binding to cells

Plasma PCI was isolated as described by Malleier et al. [13] and coupled with Cy3 (Cyanine dye with an excitation at ∼550 nm) fluorophor (Cy-3 Ab labelling kit Amersham, GE Healthcare, Uppsala, Sweden) according to the product manual. Live and apoptotic Jurkat cells (5×10^5^/tube) were washed with cold PBS (pH =  7.4) and incubated with 75 nM Annexin V-EGFP and/or 250 nM PCI-Cy3 for 1 h at room temperature (RT) in 500µl Annexin V binding buffer containing 1.8 mM Ca^2+^ (apoptosis kit, Biovision, Milpitas, CA, USA) or PBS without Ca^2+^ (pH = 7.4). After washing with the respective incubation buffer (2×500µl), cells were fixed and stored in 1% paraformaldehyde (PFA) at 4°C in the dark until FACS analysis (excitation: 488 nm, detection: Annexin V-EGFP FL-1 channel, PCI-Cy3 FL-2 channel). Differentiated U937 cells were dislodged from wells with Accutase (PAA-Laboratories, Pasching, Austria) treatment at 37°C for 30 minutes and washed. Incubation with Annexin V-FITC and PCI-Cy3 was performed in Hepes buffer (25 mM Hepes, 137 mM NaCl, 3.5 mM KCl, pH = 7.4) with or without 2.5 mM CaCl_2_.2H_2_O containing 3% BSA (PAA-Laboratories, Pasching, Austria).

### Confocal microscopy and Fluorescence Resonance Energy Transfer microscopy (FRET)

PFA-fixed samples were incubated with Hoechst 33342 dye (diluted 1∶1000, Sigma-Aldrich, St.Luis, MO, USA) for 10 minutes at RT, and mounted with Vectashield mounting medium (Vector Laboratories, Burlingame, CA, USA). Confocal laser scanning microscopy was carried out on a LSM510 (Zeiss, Jena, Germany). Further picture analysis was done with LSM Image Browser Software (Zeiss, Jena, Germany). Cells for FRET microscopy were mounted without Hoechst staining. Annexin V-FITC was used as donor and PCI-Cy3 as acceptor molecule. At least five pictures of single stained cells were acquired as control and used for correcting FRET pictures of double stained samples for the bleedthrough background of the single fluorophores. Wavelengths used for the donor alone were excitation 488 nm, emission bandpass filter 505–530 nm, for the acceptor alone were excitation 543 nm, emission bandpass filter 574–798 nm and for the FRET signal were excitation 488, emission longpass filter 560 nm. Picture corrections and calculations were done in ImageJ PixFRET (National Institutes of Health, Bethesda, MD, USA) software calculated as:

sqrtFRET  =  [FRET signal - (bleedthrough donor + bleedthrough acceptor)]/√(donor x acceptor).

### Phagocytosis assays

#### Apoptotic cells

Differentiated, adherent U937 cells were stained with 1µM Dodecyldimethylamine oxide Succinimidyl-Ester (DDAO-SE) far red (excitation 633 nm, detection FL-4 channel) and apoptotic Jurkat cells, in suspension, with 0.5µM Carboxyfluorescein Diacetat Succinimidyl Ester (CFDA-SE) cell tracker dyes (excitation 488 nm, detection FL-1 channel) according to the manufacturer's protocols (Life Technologies, Carlsbad, CA, USA). Either Jurkat cells or macrophages were incubated with 100 or 300 nM human recombinant PCI (expressed in HEK293 cell line; Novoprotein Scientific, Summit, NJ, USA), heat-treated PCI (10 Min. 100°C), human plasma purified antithrombin III (Sigma-Aldrich, St.Luis, MO, USA), or PBS buffer control, respectively, in Hepes buffer pH = 7.4 (3.1×10^6^ cells/125µl) for 1 h at 37°C. Thereafter cells were either washed twice with Hepes buffer to remove unbound protein or seeded immediately onto adherent macrophages (2.5×10^5^ macrophages in 200µl Hepes buffer containing 0.5% FBS +1.25×10^6^/50µl Ct Jurkat cells). Phagocytosis assays were conducted for 24 h at 37°C or at 4°C. To stop phagocytosis, unengulfed apoptotic cells were washed away with cold PBS and after Accutase treatment (30 min. 37°C) dislodged U937 macrophages were fixed and stored in 1% PFA at 4°C in the dark until FACS analysis. FACS recording was stopped when 5000 cells of the macrophage population (DDAO-SE positive/excitation 633, FL-4 channel) were reached. Experiments were evaluated by FCS express 4 Flow Research Edition software (DeNovo, Los Angeles, CA, USA). CFDA/DDAO-SE double positive cells represented macrophages with ingested apoptotic material. After gating for the macrophage population the phagocytosis index was determined as follows:

Phagocytosis index  = % phagocytosing macrophages (double positive cells) x mean ingested apoptotic material (median CFDA-SE fluorescence intensity of phagocytosing macrophages)

#### Polysterene microspheres

Phagocytosis experiments with fluorescent polystyrene microspheres were conducted in a similar manner. U937 cells were differentiated with 100 nM PMA for 24 h. Yellow green fluorescent microspheres with a diameter of 1µm (carboxyl modified, Sigma-Aldrich, St.Luis, MO, USA) were washed with 1 ml PBS/2×10^7^ particles and by centrifugation at 11,000 g for 15 minutes. Beads (1×10^7^ microspheres/100µl) or macrophages (250µl/well) were preincubated with buffer, 100 nM human plasma derived PCI or 100 nM AT3 in serum free medium for 1 h at 37°C. Unbound protein was washed away and 10 beads/macrophage were seeded onto differentiated macrophages and phagocytosis was conducted in cell culture medium, containing 10% FBS, for 0.5, 1, 2, 3, 4 h at 37°C or 4°C. Detachment, fixation, FACS and analysis were performed as described for phagocytosis of apoptotic cells.

#### Platelets

Phagocytosis of platelets was conducted similarly as previously described by Badrnya et al. [27]. Platelets were activated by the addition of ADP (Sigma-Aldrich, St.Luis, MO, USA) at a final concentration of 20µM for 10 minutes with or without 300 nM PCI, and dyed with CMFDA Cell Tracker green (0.1µg/ml, Life Technologies, Carlsbad, CA, USA). Excess dye was washed away with PBS and phagocytosis was carried out for 60 min with freshly isolated human blood derived mononuclear cells, isolated from the same blood donor as the platelets, at a ratio 1∶100 monocytes:platelets. Phagocytosis was stopped by fixation with 1% PFA. Surface bound platelets were stained with anti CD61-Alexa647 (BioLegend, San Diego, CA, USA) and monocytes stained with anti-CD14 PerCP (Becton Dickinson, Austria). Flow cytometric analysis was performed on an Accuri Flow Cytometer (Becton Dickinson, Austria). The amount of surface bound Cell tracker green and Alexa 647 double positive platelets on monocytes was substracted from all Cell tracker green positive platelets associated with monocytes resulting in the percentage of phagocytosed particles.

### Determination of activation markers on platelets

Isolated platelets were incubated with or without 300 nM PCI for 15 minutes, and activated by the addition of ADP (20µM) for 5 minutes. Platelet surface expression of PS and P-selectin and glycoprotein IIb/IIIa (GPIIb/IIIa) activation were detected by flow cytometry after staining with Annexin V-FITC or anti-CD62P-Alexa647 (Biolegend, San Diego, CA, USA) and PAC-1 (Becton Dickinson, Austria) for 20 minutes in binding buffer followed by fixation in 1% formaldehyd, as previously described [28].

### Fluorescence microscopy

THP-1 cells were differentiated on LabTek permanox chamber slides (Nunc, Thermo fisher scientific, Waltham, MA, USA) with 1µM PMA in full cell culture medium for 24 h at normal cell culture conditions (3.3×10^5^/well/300µl). After replacement with serum free medium, apoptotic Jurkat cells (1.65×10^6^ in 200µl serum free medium) and 50 nM PCI-Cy3 were added simultaneously and incubated for 2 h at RT. In control experiments untreated and CT Jurkat cells were incubated with PCI-Cy3 under the same conditions but in the absence of macrophages. After washing, cells were fixed in 4% PFA for 20 min, RT. Macrophages were stained with 25F9-FITC antibody (Santa Cruz, Dallas, TX, USA) and Jurkat cells with mouse anti human beta3 integrin antibody (1µg/ml) for 1 h at RT (Bioscience Research Reagents, Temecula, CA, USA) and secondary goat anti-mouse IgG Alexa488 coupled antibody (400 ng/ml) (Life Technologies, Carlsbad, CA, USA) for 45 min at RT and Hoechst (diluted 1∶1000) for 10 Minutes at RT. Fluorescence microscopy was performed on an Olympus AX70 microscope (Olympus, Tokyo, Japan), equipped with filter cubes for Hoechst: excitation 360 nm, emission 420–460 nm; FITC: excitation 450–480 nm, emission barrier filter 515; Cy3: excitation 525–560, emission 570–650 nm. Cell∧P software was used for acquisition.

### Real Time-PCR

mRNA was isolated from U937 cells (3×10^6^ cells/differentiation time point) according to the user guide instructions of the RNeasy Kit (Qiagen, Venlo, Netherlands) and transcribed into cDNA (RNase Inhibitor, dNTP mix, MuLV Reverse Transcriptase, Oligo d(T)16, MgCl_2_ and Tag buffer: Applied Biosystems, Life Technologies, Carlsbad, CA, USA) in an Eppendorf Mastercycler personal (10 min 20°C, 20 min. 42°C, 5 min 95°C). Following primers were used for RT-PCR, using 1.5µl cDNA and 7.5µl SYBR@green (Applied Biosystems, Life Technologies, Carlsbad, CA, USA) master mix and 0.375µl of each primer in a total volume of 15µl/sample:

human GAPDH

5′-GGCCTCCAAGGAGTAAGACC-3′,

3′-AGGGGTCTACATGGCAACTG-5′,

human CD14

5′-AGCCTAGACCTCAGCCACAA-3′,

3′-CTTGGCTGGCAGTCCTTTAG-5′,

human CD11b

5′-CCAGACGGAGACCAAAGTG-3′,

3′-GTCCTTGTATTGCCGCTTG-5′, (VBC Biotech, Vienna, Austria) human PCI

5′- AGGCAAGATTGTGGACTTG-3′,

3′- GTTCCGGTCCAGGAGGTAGT-5′ (Invitrogen, Life Technologies, Carlsbad, CA, USA) and run on a Real Time PCR System (Applied Biosystems StepOnePlus) using FAST PCR 96-well plate, sealed with optically clear sealing tape (Sarstedt, Nümbrecht, Germany) with the fast program (40 cycles starting 95°C 20 s, 95°C 3 s, 60°C 30 s, and a melting curve of 95°C 15 s, 60°C 60 s, 95°C 15 s).

### Detection of PCI on the cell surface

U937 cells were differentiated with 20 nM PMA or DMSO (control) at 5×10^5^ cells/ml/well for 24–72 h under normal cell culture conditions. At each time point 4 wells were washed once with cold PBS and dislodged with Accutase 200µl/well for 30 minutes. 5×10^5^cells/tube were washed twice with 500µl/tube cold Hepes buffer containing 3% BSA. Cells were blocked with 50µl buffer containing Human TruStain FcX (50µl/ml, Biolegend, San Diego, CA, USA) for 10 minutes at RT. After blocking, 50µl monoclonal anti PCI antibody (4PCI; Technoclone, Vienna, Austria) or mouse monoclonal IgG1 isotype control (R&D Systems, Minneapolis, MN, USA) were added to reach a final concentration of 30µg/ml and incubated for 1 h RT. Cells were washed twice and incubated with 50µl secondary Alexa Fluor 488 F(ab')2 fragment of goat anti-mouse IgG (H+L) (diluted 1∶1000, Invitrogen, Life Technologies, Carlsbad, CA, USA) for 45 minutes at RT. After washing, cells were fixed in 1% PFA and stored in the dark until FACS analysis. PCI detection was quantified by the mean fluorescent intensity multiplied with the percentage of Alexa 488 positive cells. The isotype control was substracted.

### Statistics

Statistics were calculated in GraphPad PRISM Software (GraphPad Software Inc., La Jolla, CA, USA) using One-way ANOVA and the Dunnett's multiple comparison, or a two tailed paired Student's T-test where indicated, *P*<0.05 was considered as significant.

## Results

### PCI binds to Camptothecin- treated cells

**Figure 1:**
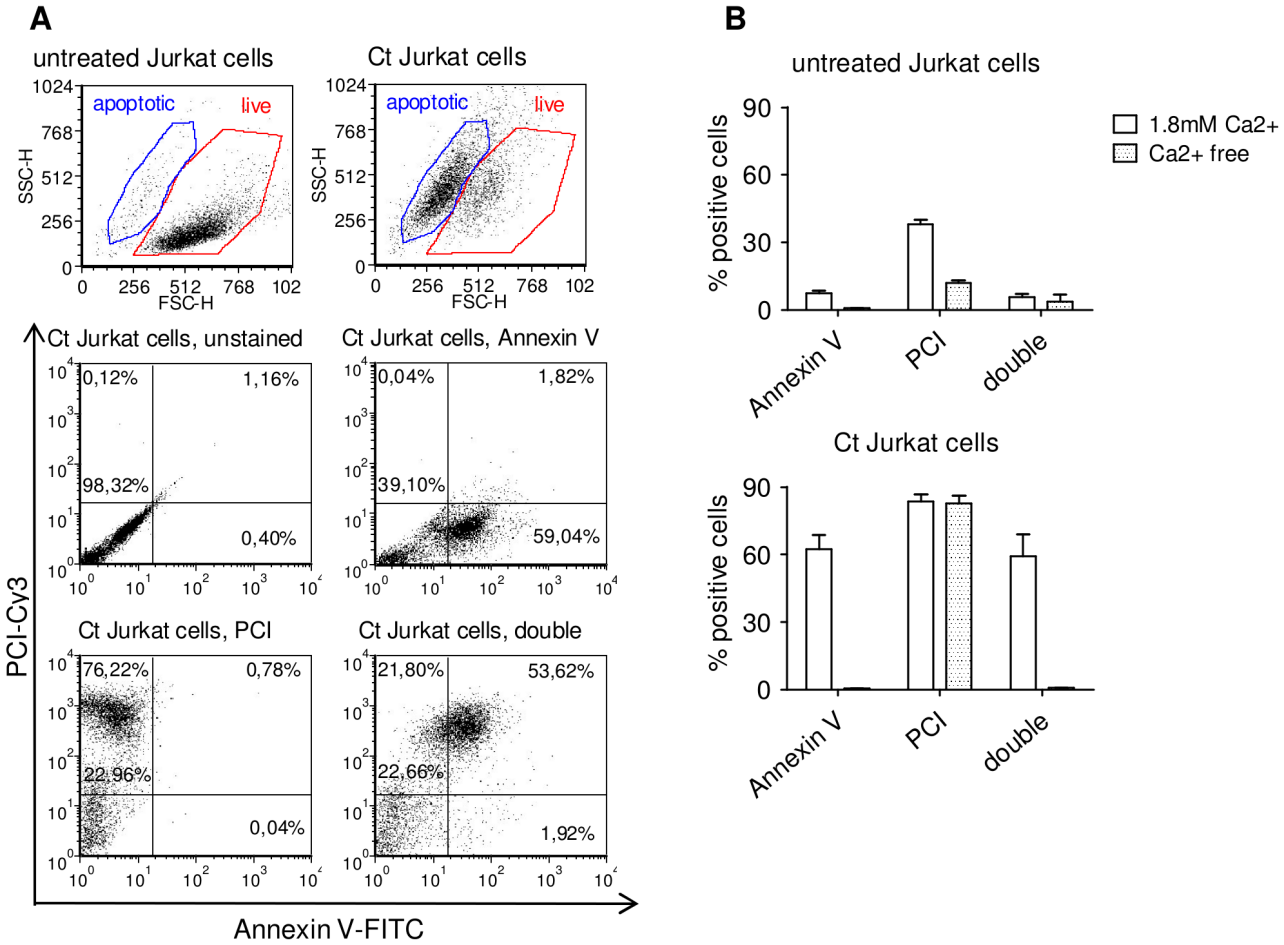
Annexin V and PCI binding to Jurkat cells. Untreated and Ct (6µM Camptothecin 24 h, in full RPMI medium at 37°C) Jurkat cells were incubated with 75 nM Annexin V-EGFP and/or 250 nM PCI-Cy3 for 1 h, RT, in Annexin V binding buffer, containing 1.8 mM Ca2+ or in PBS (Ca2+ free) and analysed by flow cytometry. Data (A) show representative dot plots from experiments performed in presence of calcium, analysed by FCS Express 4 Flow Cytometry Software (De Novo Software), bar graphs (B) represent quantification of Annexin V-, PCI- and double positive cells in Ca2+ containing (open bars) and Ca2+-free conditions (filled bars) of two independent experiments showing means and range.

In order to determine the binding of PCI, untreated and Ct Jurkat cells were incubated with PCI-Cy3 and Annexin V-EGFP, used as apoptosis marker and analysed by flow cytometry (Figure 1). By treatment of Jurkat cells with Camptothecin 50% – 80% Annexin V positive Jurkat cells were obtained (data not shown). Calcium- free conditions served as control for specific Annexin V binding. Fluorophor-linked PCI bound to a high percentage of Ct Jurkat cells, and only to a small percentage to untreated cells. Binding of PCI to Ct cells was not dependent on calcium, but the binding to untreated cells partially was (Figure 1B).

**Figure 2:**
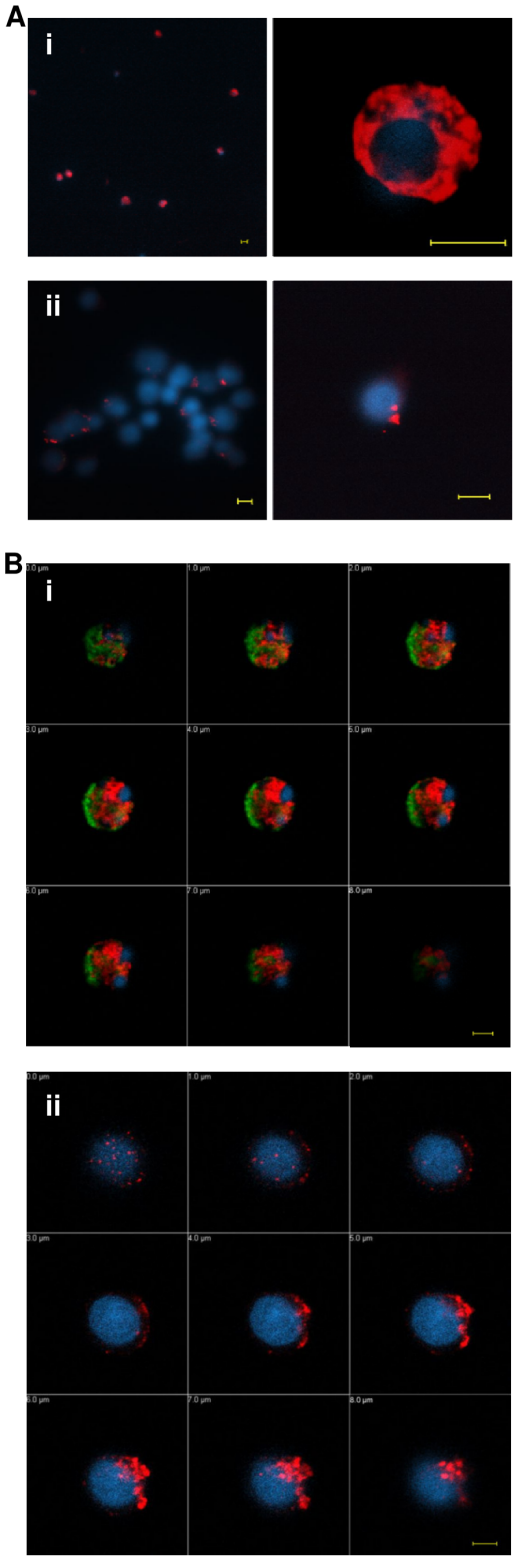
Confocal microscopy of PCI binding to untreated and Ct cells. Jurkat cells (A) and U937 cells (B) were incubated with 250 nM PCI-Cy3 (red) and in some experiments also with 75 nM Annexin V-FITC (Aii, Bi, green). Nuclear staining was done with Hoechst (blue). Confocal microscopy pictures show Ct Jurkat cells (Ai) and untreated Jurkat cells (Aii) in two different magnifications (40×, n.a. 1.3 oil objective; 100×, n.a. 1.3 oil objective). Data in (B) show z-stack galleries of 1µM slice thickness representing a Ct U937 cell (Bi) and an untreated U937 cell (Bii) (63×, n.a 1.4 oil objective). The scale bars represents a length of 5µm. Picture processing was done in LSM Image browser software (Carl Zeiss).

Confocal microscopy data of PCI binding to untreated and Ct Jurkat cells confirmed an association of PCI with Ct cells (Figure 2A). PCI was probably internalized into the cells in vesicular structures. About 10–30% of untreated Jurkat cells bound PCI. Here the binding was limited to specific spots probably on the cell surface. To prove that these results were not limited to T-lymphocytes a myeloid cell line (U937 cells) was investigated for PCI binding as well (Figure 2B). The binding to U937 cells showed a similar binding pattern, binding to Ct cells was intracellular while for the untreated cells, PCI was localised only on few cells and at distinct spots, most likely on the cell surface.

### PCI binding to U937 cells increases with macrophage differentiation

**Figure 3:**
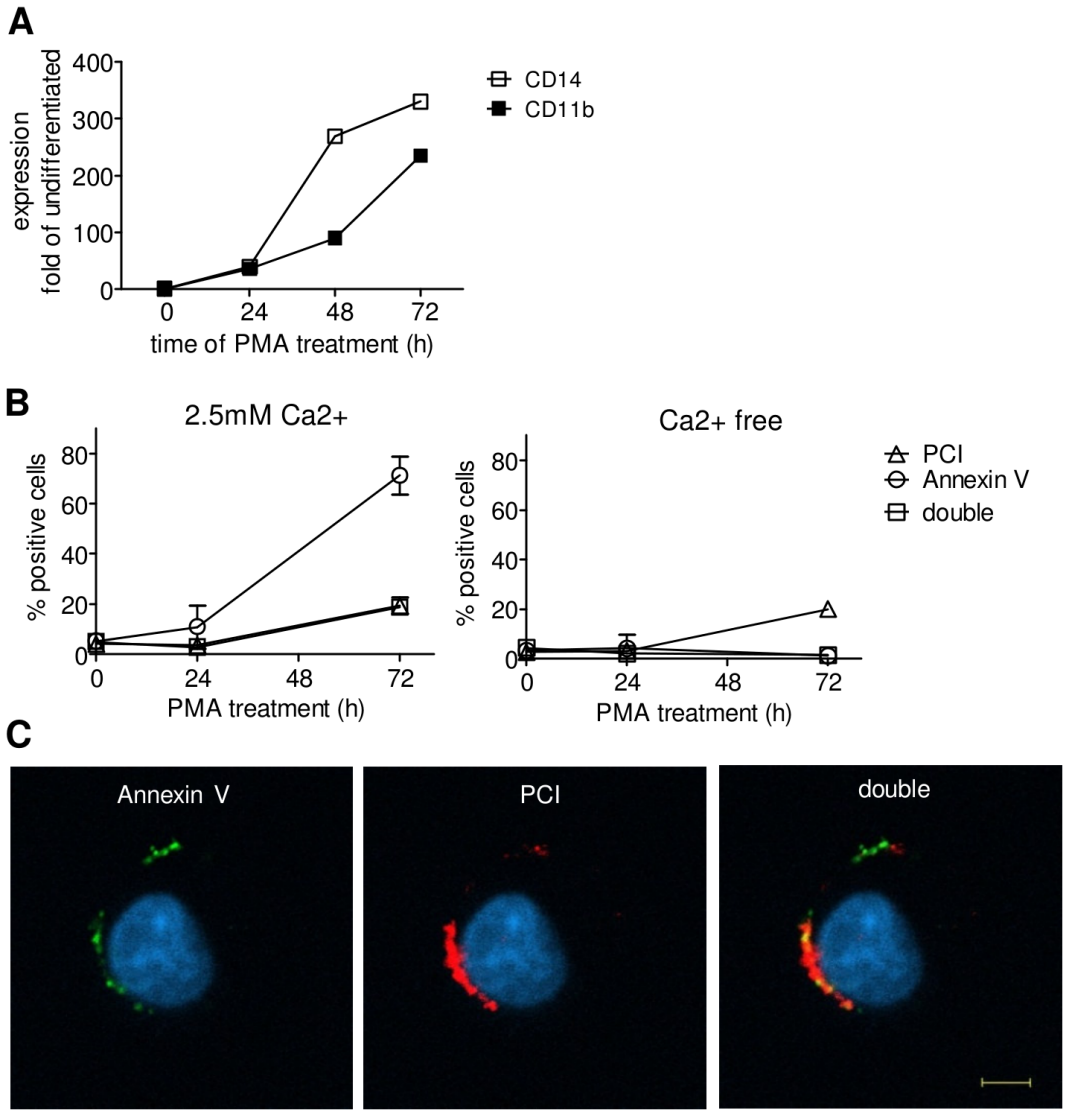
PCI binding to U937 cells is differentiation dependent. U937 cells were differentiated with 20–72 h. Panel (A): Expression of macrophage surface markers CD14 and CD11b indicating time dependent differentiation. At 0, 24 and 72 h of differentiation U937 cells were incubated with 75 nM Annexin V-FITC and/or 100 nM PCI-Cy3 for 1 h, RT, in Hepes buffer with 3% BSA±2.5 mM Ca2+. Time course data in (B) represent PCI and Annexin V binding of two independent experiments showing means and range. U937 cells, treated with PCI-Cy3 (red) and Annexin V-FITC (green) in presence of Ca2+ were analysed by confocal microscopy. Pictures (C) show 72 h differentiated U937 cells. Nuclear staining was done with Hoechst (blue). The scale bar represents a length of 5µm (63×, n.a. 1.4 oil objective).

U937 cells were differentiated to macrophage like cells by the incubation with PMA for 24 h or 72 h, respectively, and thereafter incubated with PCI-Cy3. Macrophage differentiation was confirmed by the upregulation of the macrophage markers CD14 and CD11b (Figure 3A). The amount of PCI-Cy3 positive cells increased with time of PMA treatment, as was assessed by flow cytometry. Moreover, also the amount of Annexin V-FITC positive cells increased (Figure 3B), which is in line with current literature [29]. According to confocal microscopy in Figure 3C, PCI (red) was obviously binding to the cell surface and showed also accumulation at Annexin V binding regions (green).

### PCI colocalizes with PS in close proximity on Ct cell membranes

**Figure 4:**
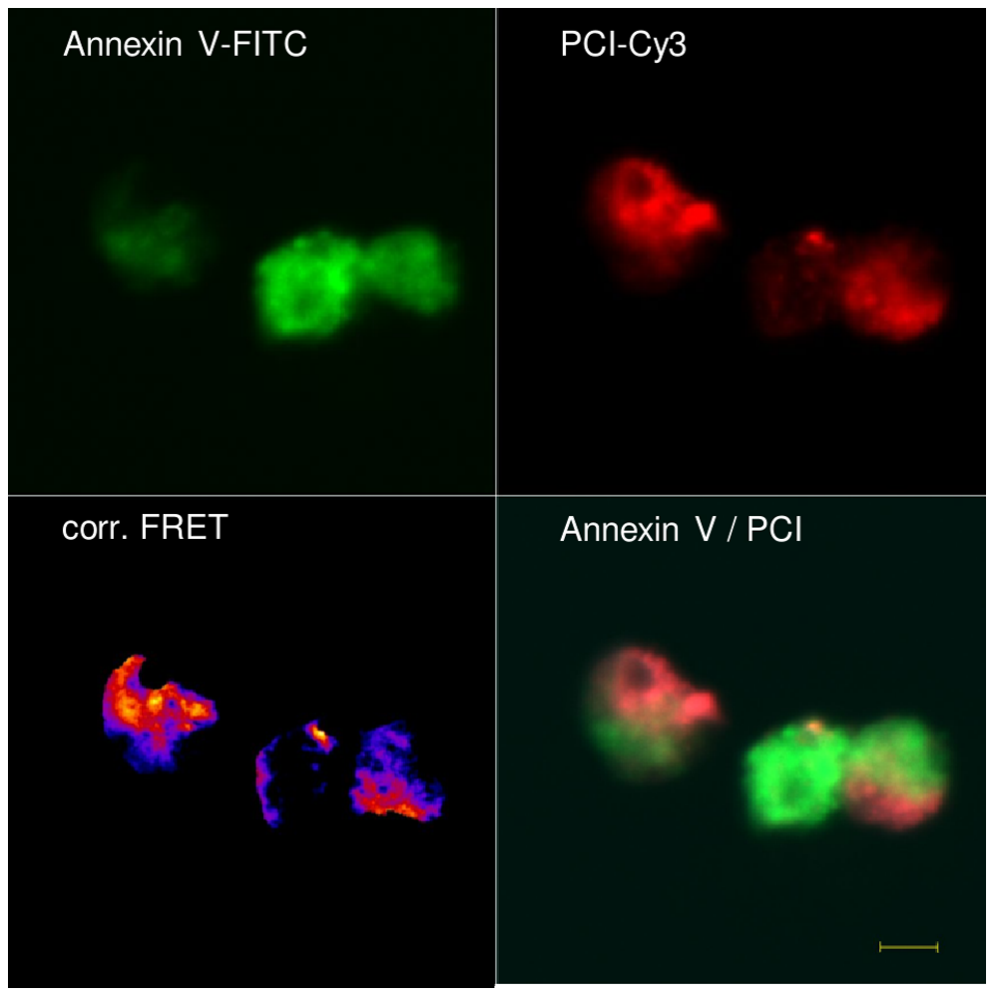
Colocalization of PCI-Cy3 and Annexin V-FITC on Ct-Jurkat cells. Ct treated Jurkat cells were incubated with 250-Cy 3 and 75 nM Annexin V-FITC and analysed for FRET signal. Calculations and corrected pictures (fire) were processed with ImageJ software using the PixFRET software as described in “Materials and Methods”, signal intensity is indicated by the color spectrum displaying intense signals in yellow. The figure shows representative pictures of two independent experiments. The scale bar shows the length of 5µm (40×, n.a. 1.3 oil objective).

In order to prove that PCI does not only bind PS in purified in vitro studies we used fluorescence resonance electron transfer microscopy (FRET) to identify a close proximity of PCI to PS on apoptotic cells. Ct Jurkat cells, which were considered as late apoptotic according to Annexin V/propidium iodide binding, were treated with Annexin V-FITC as the donor and PCI-Cy3 as the acceptor molecule of energy transfer. Corrected pictures revealed significant FRET signals (Figure 4) suggesting that PCI and Annexin V bind in close proximity to apoptotic cells.

### PCI inhibits phagocytosis of apoptotic cells

**Figure 5:**
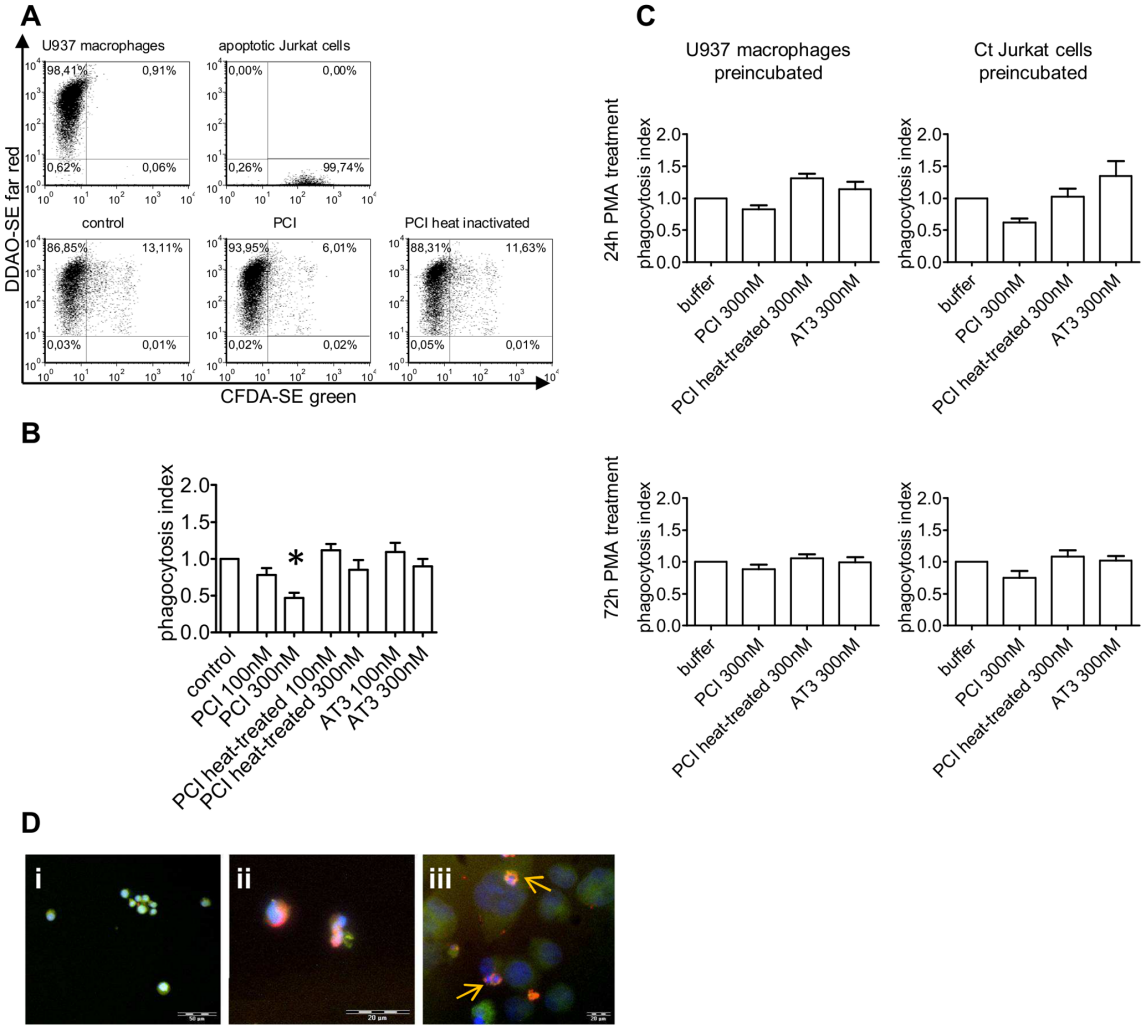
Localisation and effect of PCI in efferocytosis. Flow cytometry dot plot graphs of the phagocytosis model using 24-SE and Ct Jurkat cells stained with CFDA-SE. Double positive cells represent macrophages containing engulfed apoptotic cells (A). Bar graphs show the quantifications (as described in “Materials and Methods”) of the phagocytosis assay, after substraction of 4°C control (surface bound apoptotic cells). Jurkat cells were preincubated with the indicated concentrations of protein for 1 h at 37°C, without washing protein away, Jurkat cells were added to the U937 cells and incubated for 24 h on 37°C or 4°C. The graph represents means and SEM of three independent experiments each performed in duplicates. Statistics was evaluated by one-way ANOVA and as post test the Dunnett's multiple comparison. * = P<0.05 (B). For the results in (C) 24 h and 72 h differentiated U937 cells were used and either the macrophages or the Ct Jurkat cells were preincubated with proteins. After washing, phagocytosis was performed for 24 h at 37°C or 4°C. Data represent means and SEM after substraction of 4°C of four independent experiments, each performed in duplicates. Fluorescence microscopy pictures show untreated Jurkat cells (Di) and Ct Jurkat cells (Dii) both stained for beta3 integrin and secondary Alexa 488 antibody (green) incubated with 50 nM PCI-Cy3 (red) for 2 h. The third picture (Diii) represents 24 h PMA-differentiated THP-1 macrophages, stained with the macrophage antibody 25F9-FITC (green) and incubated with Ct Jurkat cells and PCI-Cy3 simultaneously for 2 h at RT, as described in “Materials and Methods”. Nuclear staining was done with Hoechst (blue), 10×, n.a. 0.4; 20×, n.a.0.4; 40×, n.a. 0.8. Arrows indicate apoptotic particles, covered with PCI-Cy3.

In our phagocytosis model differentiated U937 macrophages and Ct Jurkat cells were stained with different fluorophores and phagocytosis was assessed by flow cytometry (Figure 5A).

PCI inhibited phagocytosis in a dose dependent manner in an assay system where apoptotic Jurkat cells were preincubated with PCI and where PCI was present during phagocytosis (Figure 5B). At 100 nM, corresponding to the plasma concentration, PCI showed a mild, but not significant decrease, while 300 nM PCI resulted in a reduction of the phagocytosis index to half. In this model PCI influenced mainly the quantity of phagocytosing macrophages (average buffer control 17.56%±8.33), without appreciable impact on the amount of ingested material per macrophage. The heat-treated form of PCI, or antithrombin III (AT3) did not display inhibitory effects on phagocytosis under the same conditions.

Preincubation of either the apoptotic Jurkat cells or the macrophages with PCI and subsequent washing (Figure 5C) did not result in a significant effect on efferocytosis but in comparison to AT3 or heat-treated PCI, active PCI showed a mild inhibitory effect. However the tendency of phagocytosis inhibition by PCI was more prominent for preincubated Jurkat cells, than preincubated U937 macrophages, while the stage of macrophage differentiation seemed to be irrelevant.

To visualize cellular binding of PCI in the phagocytosis model, Jurkat cells (green) alone (untreated or Ct-treated) or together with THP-1 macrophages (green) were incubated with fluorophore coupled PCI (red) and analysed by fluorescence microscopy. THP-1 macrophages were used for microscopy since these cells show less clustering upon differentiation than U937 cells. The pictures revealed binding of PCI to the apoptotic Jurkat cells during phagocytosis, shown by intensely red stained small apoptotic bodies inside of larger macrophages, indicated by small arrows (Figure 5D).

### PCI inhibits the phagocytosis of activated platelets

As activated platelets expose PS on their surface [30], we investigated if PCI has an influence on phagocytosis of platelets. Therefore untreated and ADP activated platelets were coincubated with human blood derived monocytes in the presence or absence of PCI. The activation of platelets after ADP treatment was confirmed by an increase in activation markers like PS exposure (2.3±1.2 fold), glycoprotein IIb/IIIa activation (2.2±0.7 fold) and surface P-selectin expression (3.7±0.9 fold).

**Figure 6:**
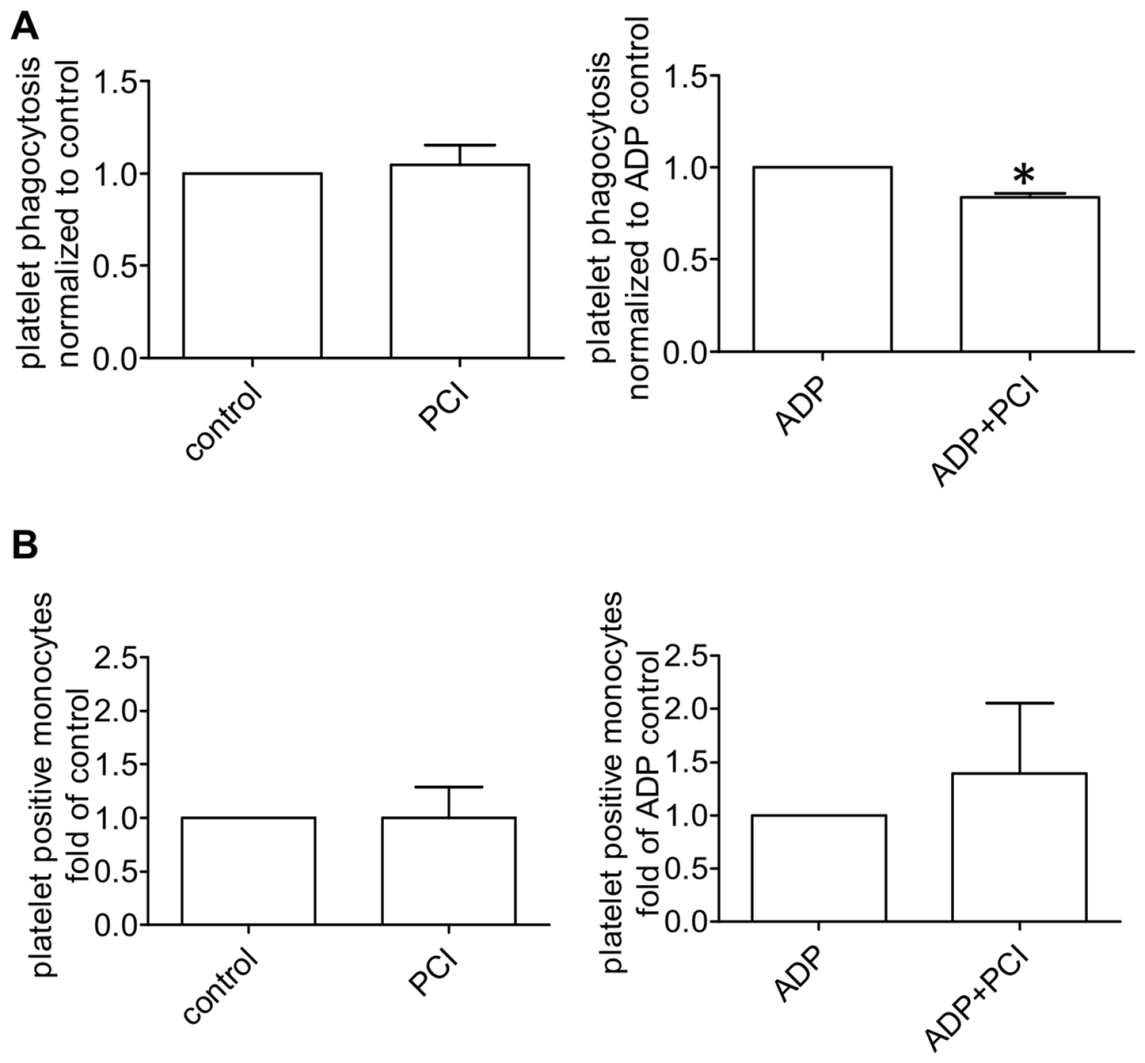
Phagocytosis of untreated and activated platelets. The graphs (A) represent phagocytosis and (B) shows surface binding of untreated and ADP (20µM) activated platelets by human blood derived monocytes with or without 300 nM PCI. Platelets were stained with CMFDA, activated with ADP and phagocytosis was conducted with monocytes for 60 minutes. Surface bound platelets, detected by CD61-Alexa647 were substracted from the total monocyte associated platelets to gain the count of phagocytosis. The bar graphs represent mean and standard deviation of 5 experiments, each performed in duplicates. Statistical significance was calculated with the two tailed paired Student's T-test,* =  P<0.05.

**Figure 7:**
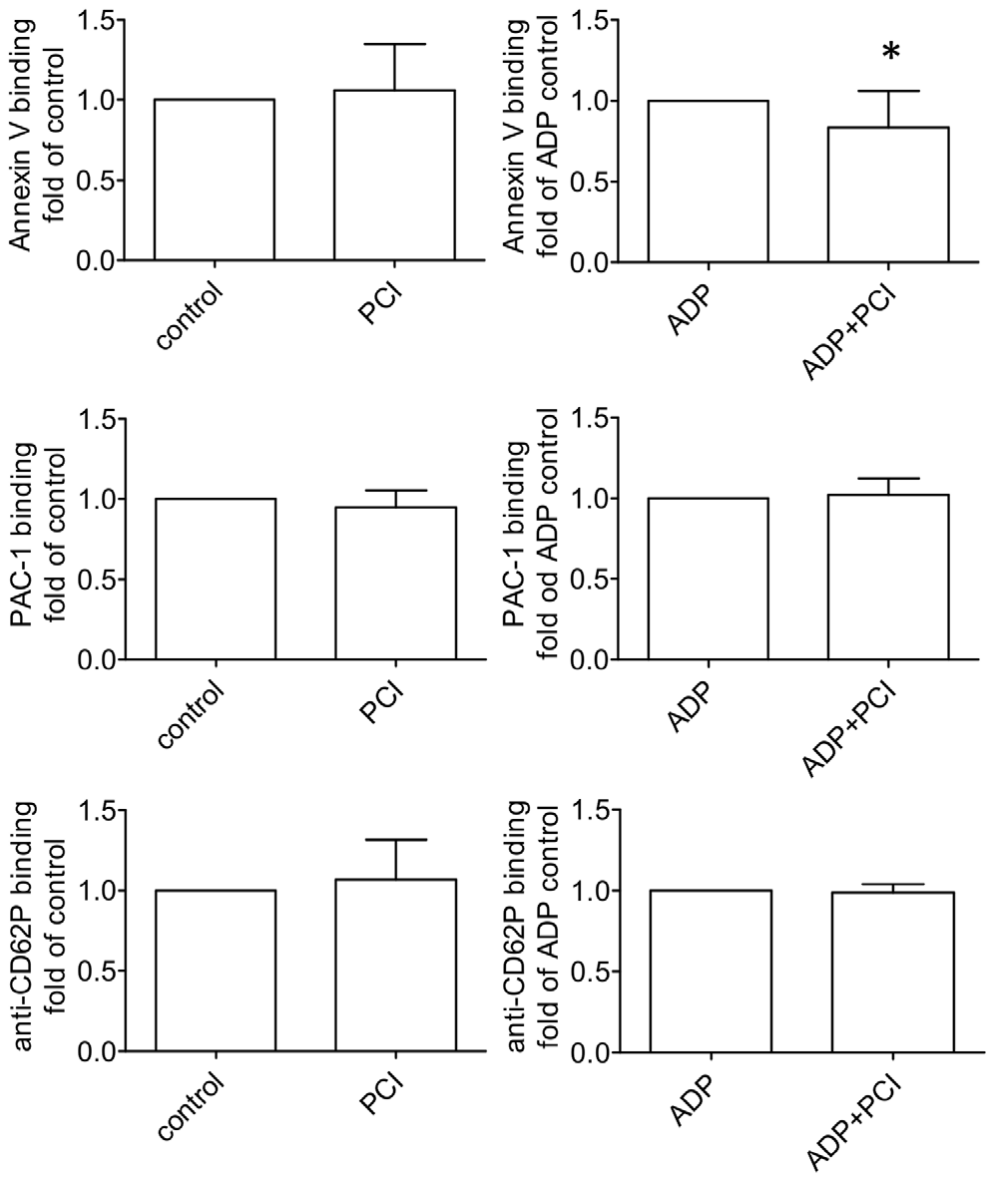
Surface markers of activated platelets. The graphs show the influence of PCI on platelet activation. Platelets were incubated with 300-FITC to detect PS surface exposure (n = 15), PAC-1 antibody to determine GPIIb/IIIa activation (n = 10) and anti-CD62P-Alexa647 for surface expression of P-selectin (n = 10). Bar graphs represent mean and standard deviation. Statistical significance was calculated with the two tailed paired Student's T-test, * =  P<0.05.

In general, the phagocytosis of platelets by monocytes isolated from the same blood donor, increased with ADP activation 3.7 times compared to untreated controls. PCI showed no effect on the phagocytosis of untreated platelets, while the phagocytosis of activated, PS exposing platelets was significantly decreased with PCI incubation compared to the control (Figure 6A). Binding of platelets to monocytes was not affected by PCI (Figure 6B). In order to rule out a difference in platelet activation in the presence of PCI, activation markers of untreated and ADP treated platelets were compared with or without PCI incubation. Annexin V binding decreased on activated platelets in comparison to the control, while the surface expression of P-selectin and GPIIb/IIIa did not show any difference (Figure 7), which suggests that PCI does not influence platelet activation itself but interacts with exposed PS.

### PCI preincubation inhibits phagocytosis of polystyrene microspheres

**Figure 8:**
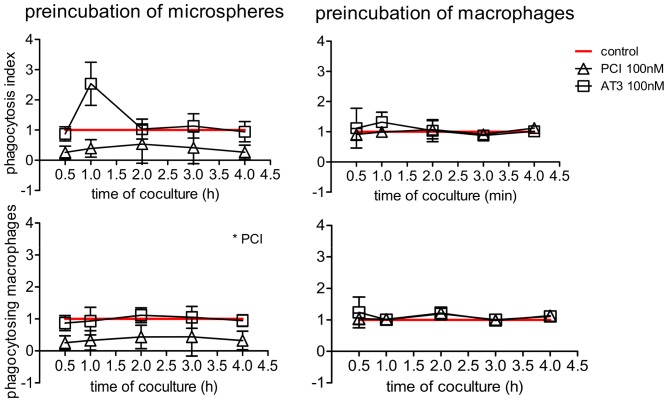
Phagocytosis inhibition of PCI binding microspheres. The graphs represent phagocytosis of microspheres by U937 macrophages. Either the microspheres or the macrophages were preincubated with buffer (control), 100 nM PCI or 100 nM AT3. After washing away unbound protein coculture was performed for indicated time periods at 37°C or 4°C. The phagocytosis index is calculated by the multiplication of the percent phagocytosing macrophages with the mean fluorescent intensity of ingested microspheres. The 4°C values were substracted and timelines were normalized to the control showing mean and SEM of three independent experiments, each performed in duplicates. Statistical significance was evaluated by one way ANOVA and the Dunnett's multiple comparison test. * = P<0.05.

To further evaluate the interaction site of PCI in the phagocytosis process phagocytosis was conducted with carboxyl modified polystyrene microspheres as target material to be engulfed by macrophages. Preincubation with proteins was conducted on macrophages or the beads. Pretreatment of the beads results in PCI binding (data not shown), which decreases the amount of phagocytosing macrophages significantly, while the ingested amount of microspheres per macrophage does not alter in relation to the control. Preincubation of macrophages with PCI or AT3 shows no effect on phagocytosis (Figure 8).

### PCI expression is upregulated during U937 macrophage differentiation

**Figure 9:**
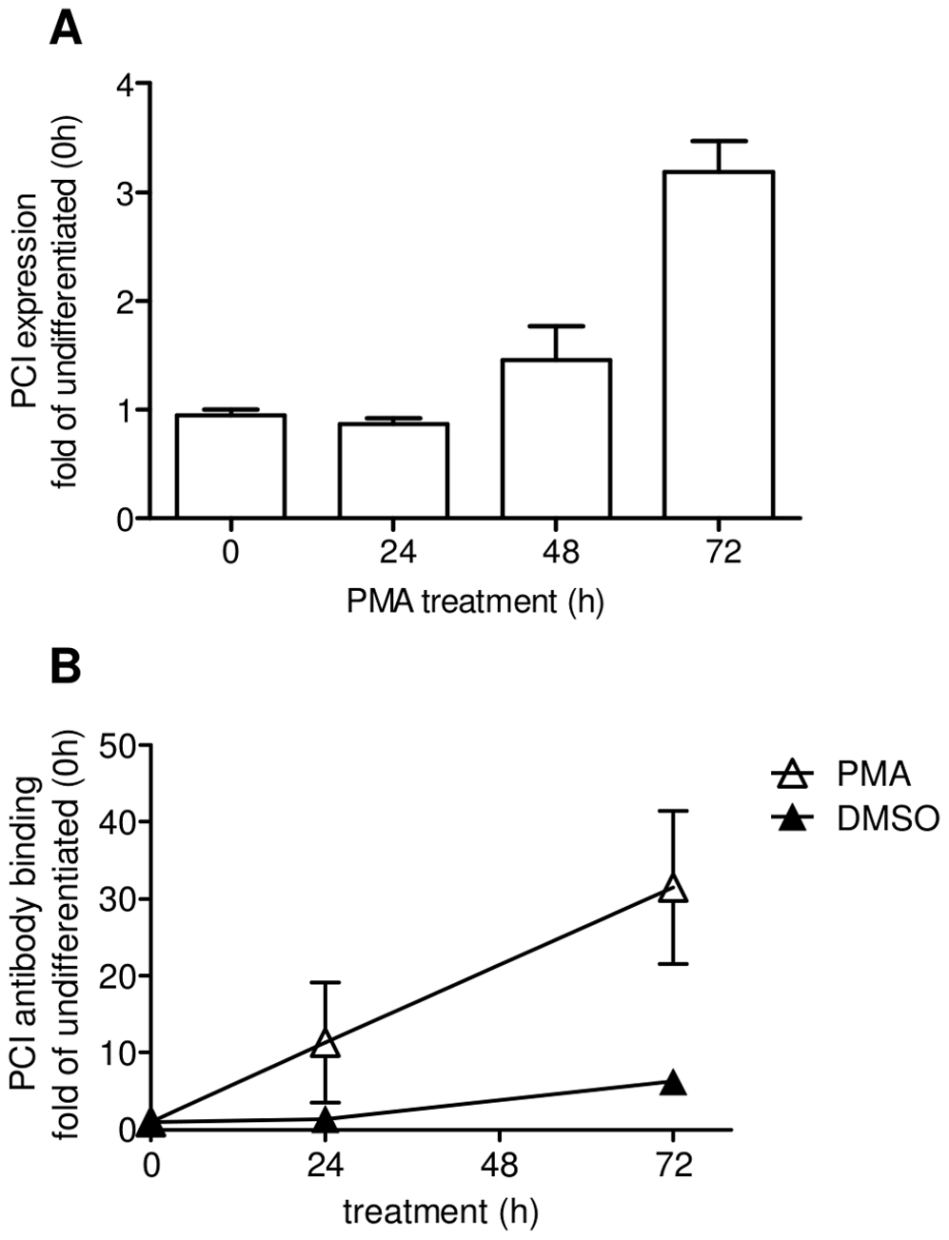
Upregulation of PCI expression during U937 macrophage differentiation. The bar graph (A) of RT PCR data shows PCI expression during differentiation of U937 cells treated with 20 nM PMA for indicated time points. The expression rate was normalized to GAPDH house-keeping gene. Data represent means and range of 2 experiments. Statistics was evaluated by one-way ANOVA and as post test the Dunnett's multiple comparison. * = P<0.05. Graph (B) shows PCI antibody binding to U937 cells during differentiation. Data represent antibody binding after substraction of the proper isotype control to the surface of PMA treated cells (open triangles) or treated with DMSO control (filled triangles) of two independent experiments, showing means and range.

PCI expression was upregulated during differentiation of U937 cells with PMA into macrophage like cells. After 72 hour treatment, PCI mRNA expression reached about three fold the expression rate of the undifferentiated U937 monocytes (Figure 9A). Also PCI-antibody binding to the cell surface of U937 cells increased with differentiation (Figure 9B).

## Discussion

PCI has been extensively studied for interactions with glycosaminoglycans and phospholipids in purified systems. Malleier et al. [13] showed the binding of PCI to negatively charged phospholipids (oxidized phosphatidylethanolamine, unoxidized and oxidized PS) and modulation of the inhibitory activity upon phospholipid binding.

In order to investigate a physiological function of this interaction, it was the intention to study the binding characteristics of PCI to cell membranes, as PS and PE are exposed on the surface of apoptotic cells. By comparing the binding of exogenously added PCI it was observed that the majority of Ct cells, considered as apoptotic cells, bound fluorophor coupled PCI, but only a small extent of untreated cells. From previous in vitro data it is known that PCI binding to phospholipids is not dependent on calcium [13], as it is the case for Annexin V [31], frequently used as apoptosis marker. PCI binding to apoptotic cells was not dependent on calcium. However, on untreated cells PCI binding was at least partially dependent on calcium, suggesting the involvement of additional or other binding partners on intact cells. From confocal microscopy it was further obvious that not only the quantity of cells binding PCI differs between Ct- and untreated cells, but also the localisation pattern. Pictures confirm that the fluorophor coupled PCI bound to almost all apoptotic cells and seemed to be internalized into the cells in vesicular structures. Baumgärtner et al. [32] propose that PCI is internalized into cells upon phosphatidylethanolamine binding. As the loss of lipid asymmetry on apoptotic cell membranes leads to an unusually high exposure of PS and PE, the internalization of PCI, visible by confocal microscopy of apoptotic cells, may result from binding to phospholipids. A similar effect was described by Kenis, showing an internalization of PS upon Annexin V binding, which consequently reduces the phagocytosis of apoptotic cells by phagocytes [20]. Live cells showed binding of PCI only at spotted sites probably located on the cell surface, although cytosolic staining cannot be excluded. Further studies are needed to investigate the binding partners at these specific punctuated sites. Our group also described the internalization and targeting of PCI to the nucleus of HL60 cells [32]. The difference between Baumgärtner's study and our present study may be explained on the one hand by different assay conditions (serum free, 37°C) and on the other hand by using a PCI directly coupled to a fluorophor, which was linked to the protein via free amino groups. This protein modification might have altered the charge and the size of the protein, or modified the accessibility of the nuclear localisation signal, resulting in impaired nuclear transport. However, the PCI binding pattern on each live and apoptotic cells seemed to be similar in T-lymphocytes (Jurkat) and in cells of the myeloid linage (U937).

PS is known as signal for the removal of apoptotic cells by phagocytes [15]. As PCI binding to and internalization into apoptotic cells was observed by FACS analysis as well as by confocal microscopy, it was intended to elucidate the role of this interaction in an in vitro model of phagocytosis. As phospholipid composition is also changing upon cell activation and PS exposure increases during U937 macrophage differentiation, binding of PCI was examined on U937 macrophages. Treatment with phorbol esters induced U937 monocytes to differentiate into the macrophage linage. PCI binding during differentiation was studied on U937 cells up to 72 hours and revealed an increase in PCI binding cells with macrophage maturation. According to literature also the amount of Annexin V binding cells increases with differentiation [29]. Strikingly, binding of PCI to differentiated live U937 was not dependent on calcium. As this was the same for apoptotic but not for live Jurkat cells, this might point out in an increased binding to phospholipids on U937 macrophages. The question is, why there were much more Annexin V binding cells than PCI binding cells within the differentiated macrophage population, while on apoptotic cells the amount was very similar. We can show that endogenous PCI expression and surface exposure increases with macrophage differentiation. It is therefore also possible that PS is partially occupied by endogenous PCI and no longer available for PCI-Cy3. Binding and PCI localization was studied by confocal microscopy and clearly showed PCI on the surface of differentiated macrophages. Even an accumulation at Annexin V positive sites and colocalization was obvious, suggesting binding of PCI to PS.

Apart from PCI binding to PS exposing cells, further proof was needed to identify an interaction of PCI with PS on cell membranes. FRET analysis using FITC coupled Annexin V as donor molecule and PCI linked to Cy3 as acceptor, revealed clear FRET signals, suggesting a very close proximity between Annexin V and PCI. Therefore the binding partners of each protein must be very close. Competition experiments (data not shown) with Annexin V and PCI on apoptotic cells showed that the two proteins do not compete for the same binding site, therefore the positive FRET signals suggest binding to different epitopes. PS is known to be expressed as “patch”-like structures at the surface, probably together with a panel of other apoptosis signals, meaning that a dense area of PS lipids might also bind PCI and Annexin V simultaneously but to separate PS molecules, bringing both proteins in close connection [33]. PCI was probably covered all over with the Cy3 fluorophor as the dye was coupled to all free amino-groups on the protein. Hence, it seems likely that the close proximity of the molecules, even if not specifically interacting witch each other, resulted in a positive FRET signal. Confocal microscopy showed only little colocalization of PCI and Annexin V, which might be explained by higher affinity of PCI for PE and internalization, of PCI. By accumulation of PCI at PE rich sites, fainter stainings at Annexin V binding sites might be overseen according to acquisition and resolution parameter adjustments which were adapted to areas of highest signals. Therefore FRET signals prove an overlap of PCI and Annexin V at various sites of apoptotic cells, which is not easily recognized by two color confocal microscopy.

Having shown binding of PCI to both interaction partners (CT Jurkat cells as well as differentiated U937 macrophages), it seemed important to define the impact of PCI binding on the process of apoptotic cell phagocytosis. We can show that PCI inhibits phagocytosis in a dose dependent manner, which is in line with another member of the Serpin family, plasminogen activator inhibitor-1, which has been shown to inhibit efferocytosis of neutrophils [34]. Furthermore the effect of PCI was not dependent on the degree of macrophage differentiation. To show that inhibition of phagocytosis was not caused by a general protein effect, heat-treated PCI and another protein of the serpin family were studied. Heat denatured PCI did not reveal such an effect, which suggests either the importance of protease inhibitory activity for phagocytosis inhibition or altered phospholipid binding of the denatured protein. Antithrombin, another serpin and member of the same phylogenetically related class as PCI, did not show any effect on phagocytosis, which is consistent with previous in vitro data showing no binding of AT3 to PS [13].

The exact mode of interference of PCI in this system still needs to be elucidated, but the fact that efferocytosis inhibition seemed to be dependent on binding of PCI to apoptotic cells, the hypothesis of covering phagocytosis signals like PS, as it was already published for High mobility group protein-1 [21], seems very likely. Also Annexin V binding to and internalization of PS on apoptotic cell surfaces was reported to inhibit efferocytosis. As PCI also shows accumulation in endocytic vesicles in apoptotic cells, according to confocal microscopy (Figure 2), a similar mechanism might be considered for PCI [20].

Experiments, investigating the influence of PCI on the phagocytosis of untreated and activated human blood derived platelets by monocytes showed similar effects. PCI decreased phagocytosis of PS exposing, activated platelets, but does not influence phagocytosis of untreated platelets. Furthermore, our results clearly show no difference in platelet binding to monocytes. Therefore it seems very likely, that PCI is not affecting the binding step, but probably influences the internalization step during the course of phagocytosis. In order to rule out a difference in platelet activation in the presence of PCI, different activation markers were measured on the surface of the platelets with and without PCI. Previous studies on neutrophil-mediated phagocytosis of platelets revealed that while P-selectin is important for platelet adhesion to neutrophils, surface expression of PS is essential for phagocytosis of platelets [35]. In our experiments we did not observe any effect of PCI on surface expression of platelet P-selectin, which might explain the unchanged (rather slightly upregulated) formation of platelet-monocyte aggregates. However, PCI decreased the surface expression of PS on the platelet surface thereby potentially interfering with platelet uptake by monocytes. One hypothesis to explain this phenomenon, in line with our observations on apoptotic cells, is internalization of PS-PCI resulting in decreased amounts of surface exposed PS, available for Annexin V binding. Although the exact interaction mechanism of PCI in phagocytosis needs to be clarified, the internalization of PS upon PCI binding could well explain similar monocyte binding rates but decreased phagocytosis.

The physiological relevance of PCI in inhibiting efferocytosis might be described as balancing factor. PS serves as a signal for phagocytosis and PS exposure occurs also on activated platelets, on activated lymphocytes and on mature macrophages [29], [30], [36]. Therefore it seems reasonable that PS alone is not sufficient as a phagocytosis signal but that the interaction with other expressed surface markers and linking molecules defines the fate of a cell. As PCI is present in almost every body fluid, the inhibition of such an important process as apoptotic cell removal by PCI seems pointless. The serpin may rather act as a balancing regulator, preventing the phagocytosis of functional cells, exposing PS for other reasons than apoptosis. In addition, PCI binding to surfaces without PS, also inhibited phagocytosis, as it was shown in experiments with polystyrene microspheres. Here the presence of PCI on the beads causes a decline in the amount of phagocytosing macrophages. This might indicate that PCI alone acts as a phagocytosis inhibitor and the lipid surface composition of activated and apoptotic cells together with local PCI expression levels contribute to balancing immunemodulatory processes.

Real Time PCR data on the differentiation of U937 cells revealed an increase in PCI expression with degree of differentiation. The reason for this increase is not yet clear and needs further study but might indicate locally and tissue specific higher PCI levels compared to plasma concentration. Therefore the application of more than 100 nM (plasma concentration) PCI is assumed as reasonable and physiological relevant amount. According to previous publications, pointing at the stimulation of pathogen removal by PCI, this upregulation of PCI in immune cells might not only relate to phagocytosis of apoptotic cells but, indicates a much more expansive, and as yet undefined, role of PCI in immunity and inflammation [32], [37].

## References

[1] Suzuki K, Deyashiki Y, Nishioka J, Kurachi K, Akira M, et al (1987) Characterization of a cDNA for human protein C inhibitor. A new member of the plasma serine protease inhibitor superfamily. J Biol Chem 262: 611–616. 3027058

[2] Marlar RA, Griffin JH (1980) Deficiency of protein C inhibitor in combined factor V/VIII deficiency disease. J Clin Invest 66: 1186–1189. 6253526 10.1172/JCI109952 PMC371561

[3] Meijers JC, Kanters DH, Vlooswijk RA, van Erp HE, Hessing M, et al (1988) Inactivation of human plasma kallikrein and factor XIa by protein C inhibitor. Biochemistry 27: 4231–4237. 2844223 10.1021/bi00412a005

[4] España F, Berrettini M, Griffin JH (1989) Purification and characterization of plasma protein C inhibitor. Thromb Res 55: 369–384. 2551064 10.1016/0049-3848(89)90069-8

[5] Carroll VA, Griffiths MR, Geiger M, Merlo C, Furlan M, et al (1997) Plasma protein C inhibitor is elevated in survivors of myocardial infarction. Arterioscler Thromb Vasc Biol 17: 114–118. 9012645 10.1161/01.atv.17.1.114

[6] Nilsson G, Strandberg K, Astermark J, Vernersson E, Stenflo J, et al (2007) The APC-PCI complex concentration predicts outcome of aortic surgery. Thromb Res 120: 237–244. 17141298 10.1016/j.thromres.2006.10.004

[7] Watanabe R, Wada H, Sakakura M, Mori Y, Nakasaki T, et al (2000) Plasma levels of activated protein C-protein C inhibitor complex in patients with hypercoagulable states. Am J Hematol 65: 35–40. 10936861 10.1002/1096-8652(200009)65:1<35::aid-ajh6>3.0.co;2-1

[8] Suzuki K, Nishioka J, Kusumoto H, Hashimoto S (1984) Mechanism of inhibition of activated protein C by protein C inhibitor. J Biochem 95: 187–195. 6323392 10.1093/oxfordjournals.jbchem.a134583

[9] Kuhn (1990) Elucidating the structural chemistry of glycosaminoglycan recognition by protein C inhibitor. 10.1073/pnas.87.21.8506 PMC54985 2172989

[10] Shen L, Villoutreix BO, Dahlbäck B (1999) Involvement of Lys 62(217) and Lys 63(218) of human anticoagulant protein C in heparin stimulation of inhibition by the protein C inhibitor. Thromb Haemost 82: 72–79. 10456457

[11] Shirk RA, Elisen MG, Meijers JC, Church FC (1994) Role of the H helix in heparin binding to protein C inhibitor. J Biol Chem 269: 28690–28695. 7961820

[12] Réhault SM, Zechmeister-Machhart M, Fortenberry YM, Malleier J, Binz NM, et al (2005) Characterization of recombinant human protein C inhibitor expressed in Escherichia coli. Biochim Biophys Acta 1748: 57–65. 15752693 10.1016/j.bbapap.2004.12.003

[13] Malleier JM, Oskolkova O, Bochkov V, Jerabek I, Sokolikova B, et al (2007) Regulation of protein C inhibitor (PCI) activity by specific oxidized and negatively charged phospholipids. Blood 109: 4769–4776. 17332248 10.1182/blood-2006-09-046953

[14] Fadok VA, Voelker DR, Campbell PA, Cohen JJ, Bratton DL, et al (1992) Exposure of phosphatidylserine on the surface of apoptotic lymphocytes triggers specific recognition and removal by macrophages. J Immunol 148: 2207–2216. 1545126

[15] Fadok VA, de Cathelineau A, Daleke DL, Henson PM, Bratton DL (2001) Loss of phospholipid asymmetry and surface exposure of phosphatidylserine is required for phagocytosis of apoptotic cells by macrophages and fibroblasts. J Biol Chem 276: 1071–1077. 10986279 10.1074/jbc.M003649200

[16] Greenberg ME, Sun M, Zhang R, Febbraio M, Silverstein R, et al (2006) Oxidized phosphatidylserine-CD36 interactions play an essential role in macrophage-dependent phagocytosis of apoptotic cells. J Exp Med 203: 2613–2625. 17101731 10.1084/jem.20060370 PMC2118161

[17] Kobayashi N, Karisola P, Peña-Cruz V, Dorfman DM, Jinushi M, et al (2007) TIM-1 and TIM-4 glycoproteins bind phosphatidylserine and mediate uptake of apoptotic cells. Immunity 27: 927–940. 18082433 10.1016/j.immuni.2007.11.011 PMC2757006

[18] Hanayama R, Tanaka M, Miwa K, Shinohara A, Iwamatsu A, et al (2002) Identification of a factor that links apoptotic cells to phagocytes. Nature 417: 182–187. 12000961 10.1038/417182a

[19] Anderson HA, Maylock CA, Williams JA, Paweletz CP, Shu H, et al (2003) Serum-derived protein S binds to phosphatidylserine and stimulates the phagocytosis of apoptotic cells. Nat Immunol 4: 87–91. 12447359 10.1038/ni871

[20] Kenis H, van Genderen H, Deckers NM, Lux PA, Hofstra L, et al (2006) Annexin A5 inhibits engulfment through internalization of PS-expressing cell membrane patches. Exp Cell Res 312: 719–726. 16380116 10.1016/j.yexcr.2005.11.023

[21] Liu G, Wang J, Park YJ, Tsuruta Y, Lorne EF, et al (2008) High mobility group protein-1 inhibits phagocytosis of apoptotic neutrophils through binding to phosphatidylserine. J Immunol 181: 4240–4246. 18768881 10.4049/jimmunol.181.6.4240 PMC2597447

[22] Zhang WJ, Hufnagl P, Binder BR, Wojta J (2003) Antiinflammatory activity of astragaloside IV is mediated by inhibition of NF-kappaB activation and adhesion molecule expression. Thromb Haemost 90: 904–914. 14597987 10.1160/TH03-03-0136

[23] Huber J, Boechzelt H, Karten B, Surboeck M, Bochkov VN, et al (2002) Oxidized cholesteryl linoleates stimulate endothelial cells to bind monocytes via the extracellular signal-regulated kinase 1/2 pathway. Arterioscler Thromb Vasc Biol 22: 581–586. 11950694 10.1161/01.atv.0000012782.59850.41

[24] Schuster C, Fernbach N, Rix U, Superti-Furga G, Holy M, et al (2007) Selective serotonin reuptake inhibitors–a new modality for the treatment of lymphoma/leukaemia? Biochem Pharmacol 74: 1424–1435. 17709099 10.1016/j.bcp.2007.07.017

[25] Lemarie A, Morzadec C, Mérino D, Micheau O, Fardel O, et al (2006) Arsenic trioxide induces apoptosis of human monocytes during macrophagic differentiation through nuclear factor-kappaB-related survival pathway down-regulation. J Pharmacol Exp Ther 316: 304–314. 16174796 10.1124/jpet.105.092874

[26] Zhao KW, Li X, Zhao Q, Huang Y, Li D, et al (2004) Protein kinase Cdelta mediates retinoic acid and phorbol myristate acetate-induced phospholipid scramblase 1 gene expression: its role in leukemic cell differentiation. Blood 104: 3731–3738. 15308560 10.1182/blood-2004-04-1630

[27] Badrnya S, Schrottmaier WC, Kral JB, Yaiw KC, Volf I, et al (2014) Platelets mediate oxidized low-density lipoprotein-induced monocyte extravasation and foam cell formation. Arterioscler Thromb Vasc Biol 34: 571–580. 24371083 10.1161/ATVBAHA.113.302919

[28] Assinger A, Koller F, Schmid W, Zellner M, Babeluk R, et al (2010) Specific binding of hypochlorite-oxidized HDL to platelet CD36 triggers proinflammatory and procoagulant effects. Atherosclerosis 212: 153–160. 20684828 10.1016/j.atherosclerosis.2010.05.010

[29] Callahan MK, Halleck MS, Krahling S, Henderson AJ, Williamson P, et al (2003) Phosphatidylserine expression and phagocytosis of apoptotic thymocytes during differentiation of monocytic cells. J Leukoc Biol 74: 846–856. 12960250 10.1189/jlb.0902433

[30] Bevers EM, Comfurius P, Zwaal RF (1983) Changes in membrane phospholipid distribution during platelet activation. Biochim Biophys Acta 736: 57–66. 6418205 10.1016/0005-2736(83)90169-4

[31] Andree HA, Reutelingsperger CP, Hauptmann R, Hemker HC, Hermens WT, et al (1990) Binding of vascular anticoagulant alpha (VAC alpha) to planar phospholipid bilayers. J Biol Chem 265: 4923–4928. 2138622

[32] Baumgärtner P, Geiger M, Zieseniss S, Malleier J, Huntington JA, et al (2007) Phosphatidylethanolamine critically supports internalization of cell-penetrating protein C inhibitor. J Cell Biol 179: 793–804. 18025309 10.1083/jcb.200707165 PMC2080921

[33] Kenis H, van Genderen H, Bennaghmouch A, Rinia HA, Frederik P, et al (2004) Cell surface-expressed phosphatidylserine and annexin A5 open a novel portal of cell entry. J Biol Chem 279: 52623–52629. 15381697 10.1074/jbc.M409009200

[34] Park YJ, Liu G, Lorne EF, Zhao X, Wang J, et al (2008) PAI-1 inhibits neutrophil efferocytosis. Proc Natl Acad Sci U S A 105: 11784–11789. 18689689 10.1073/pnas.0801394105 PMC2575264

[35] Maugeri N, Rovere-Querini P, Evangelister V, Covino C, Capobianco A, et al (2009) Neutrophils phagocytose activated platelets in vivo: a phosphatidylserien, P-selectin, and {beta}2 integrin dependent cell clearance program. Blood 113: 5254–65. 19264679 10.1182/blood-2008-09-180794

[36] Elliott JI, Surprenant A, Marelli-Berg FM, Cooper JC, Cassady-Cain RL, et al (2005) Membrane phosphatidylserine distribution as a non-apoptotic signalling mechanism in lymphocytes. Nat Cell Biol 7: 808–816. 16025105 10.1038/ncb1279

[37] Malmström E, Mörgelin M, Malmsten M, Johansson L, Norrby-Teglund A, et al (2009) Protein C inhibitor–a novel antimicrobial agent. PLoS Pathog 5: e1000698. 20019810 10.1371/journal.ppat.1000698 PMC2788422

